# A holistic approach to integrating patient, family, and lived experience voices in the development of the BrainHealth Databank: a digital learning health system to enable artificial intelligence in the clinic

**DOI:** 10.3389/frhs.2023.1198195

**Published:** 2023-10-19

**Authors:** Joanna Yu, Nelson Shen, Susan Conway, Melissa Hiebert, Benson Lai-Zhao, Miriam McCann, Rohan R. Mehta, Morena Miranda, Connie Putterman, Jose Arturo Santisteban, Nicole Thomson, Courtney Young, Lina Chiuccariello, Kimberly Hunter, Sean Hill

**Affiliations:** ^1^ Krembil Centre for Neuroinformatics, Centre for Addiction and Mental Health, Toronto, ON, Canada; ^2^ Health and Technology, Vector Institute for Artificial Intelligence, Toronto, ON, Canada; ^3^ Institute of Health Policy, Management and Evaluation, University of Toronto, Toronto, ON, Canada; ^4^ AMS Healthcare, Toronto, ON, Canada; ^5^ Centre for Addictions and Mental Health, Toronto, ON, Canada; ^6^ CanChild, Hamilton, ON, Canada; ^7^ CHILD-BRIGHT Network, Montreal, QC, Canada; ^8^ Kids Brain Health Network, Burnaby, ON, Canada; ^9^ Province of Ontario Neurodevelopmental (POND) Network, Toronto, ON, Canada; ^10^ Department of Psychiatry, University of Toronto, Toronto, ON, Canada

**Keywords:** patient and family engagement, lived experience engagement, mental health, digital health technology, artificial intelligence, machine learning, learning health system

## Abstract

Artificial intelligence, machine learning, and digital health innovations have tremendous potential to advance patient-centred, data-driven mental healthcare. To enable the clinical application of such innovations, the Krembil Centre for Neuroinformatics at the Centre for Addiction and Mental Health, Canada's largest mental health hospital, embarked on a journey to co-create a digital learning health system called the BrainHealth Databank (BHDB). Working with clinicians, scientists, and administrators alongside patients, families, and persons with lived experience (PFLE), this hospital-wide team has adopted a systems approach that integrates clinical and research data and practices to improve care and accelerate research. PFLE engagement was intentional and initiated at the conception stage of the BHDB to help ensure the initiative would achieve its goal of understanding the community's needs while improving patient care and experience. The BHDB team implemented an evolving, dynamic strategy to support continuous and active PFLE engagement in all aspects of the BHDB that has and will continue to impact patients and families directly. We describe PFLE consultation, co-design, and partnership in various BHDB activities and projects. In all three examples, we discuss the factors contributing to successful PFLE engagement, share lessons learned, and highlight areas for growth and improvement. By sharing how the BHDB navigated and fostered PFLE engagement, we hope to motivate and inspire the health informatics community to collectively chart their paths in PFLE engagement to support advancements in digital health and artificial intelligence.

## 1. Introduction

Rapid advancements in digital health technologies and artificial intelligence/machine learning (AI/ML) have created tremendous potential for transformative change in healthcare. Applying AI/ML algorithms to large datasets generated by digital health technologies extends potential benefits beyond care provision, including healthcare planning, treatment, prevention, public health, and disease progression (1). In mental health, AI can improve diagnosis and predict risk, support remote monitoring, and enable access to more personalized and de-stigmatized treatment forms (2–5).

Despite the transformative potential, there have been varying degrees of success with implementing digital health and AI, as many initiatives faced barriers that hindered acceptance, uptake, and adoption. Barriers are commonly socio-technological, where socio-cultural factors affect individual perceptions, acceptance, use, and adoption of technology (6). Failed implementations of large-scale health data initiatives highlighted the importance of public perspectives on how data is used and managed (7). Given the complexity of AI initiatives, meaningful engagement is critical to improving the systems' fairness, accountability, and transparency (8). Furthermore, the limited evidence on the effectiveness of digital health and AI/ML for mental health makes public engagement increasingly important, as there are potential unintended privacy and health implications to any design decisions with its development. Dialogues on values, needs, and nuanced insights derived through lived experiences are critical to inform decisions about the governance, design and implementation of these data initiatives (9, 10). As such, sustained engagement from the initiative's outset would ensure meaningful contributions in shaping its development and governance in a trustworthy, ethical, and acceptable manner (11–15).

Engagement can come in many forms and range in degree of involvement (16, 17). The Carman Patient Engagement Framework (18) suggests the spectrum ranges from “consultation” to “involvement” to partnership (or shared leadership), where patients, families, and people with lived experience (PFLE) become increasingly involved, and their perspectives have a more significant impact on organizational decision-making. PFLE engagement guides and toolkits have commonly suggested the need for organizational readiness and commitment to engage; clear visions and opportunities for engagement; a common understanding amongst all stakeholders; accommodations that promote a safe, inclusive environment, evaluation of engagement, and mechanisms to report feedback (19). While these frameworks inform approaches, they may have limited transferability across contexts, requiring organizations or initiatives to adapt approaches to suit their context (20, 21). This perspective article shares our approach and reflection on achieving meaningful PFLE engagement in developing a complex, large-scale digital health care and research initiative called the BrainHealth Databank (BHDB). The BHDB is intended to serve as the foundation for AI/ML applications at the Centre for Addiction and Mental Health (CAMH).

### 1.1. BrainHealth Databank at the centre for addiction and mental health

The BHDB is a hospital-wide initiative that advances patient-centred, data-driven care at CAMH — Canada's largest mental health teaching hospital and a leading research institute (22). The BHDB is a repository of numerous data types from various sources (e.g., sleep and physical activity, blood samples and brain images, clinical assessments, etc.). As the foundation for a digital health learning system, the BHDB will leverage clinical data to inform research and research data to inform care. Its objective is to improve our understanding of the mental illness of individuals and broader populations to accelerate the ability to deliver personalized care. This transformative initiative is the first in the Canadian mental health context (23).

As a research Centre focused on developing clinical applications of AI/ML and computational modelling, CAMH's Krembil Centre for Neuroinformatics (KCNI) led the development of the BHDB by building upon CAMH's strategic investments in core infrastructure. This includes the Cerner Millennium Electronic Health Record (EHR) and CAMH Neuroinformatics Platform to support multimodal research studies (24, 25). To support care delivery, CAMH clinics have developed and implemented evidence-based integrated care pathways that utilize measurement-based care (MBC) to monitor patient progress and inform clinical decisions (26–28).

The BHDB was developed in collaboration with clinicians, scientists, data engineers, clinical application specialists, privacy officers, legal counsel, ethicists, hospital administrators, and PFLEs to leverage their expertise to inform the various aspects of development and implementation. The first phase of the BHDB enhanced digital technology infrastructure by adding new capabilities, including electronic self-assessment capture and patient trajectory visualization, which have already influenced care. Digital MBC allows patients to complete assessments on their own device or clinic tablet before appointments resulting in efficiencies and increased patient flow (29). It has enabled the generation of real-time visual displays of patient treatment journeys, allowing clinicians to assess patients' progress quickly.

To better understand patient trajectories, the BHDB is enriching EHR treatment trajectory data by integrating the collection of research samples and daily activity and sleep data from wearable devices. The accumulated rich, integrated real-world clinical and research data is ideal for future AI/ML applications. Study participants can consent to donate their data to the BHDB for secondary use by other researchers. A patient-facing version of this dashboard is in development.

## 2. Engaging patients, families, and people with lived experience

Given that the core BHDB goal is to improve patient-centred, data-driven mental health, there was a recognition that PFLE engagement is critical during the initial planning stages to ensure the foundation of the BHDB was patient-centred and based on community needs. CAMH and KCNI's commitment to meaningful PFLE engagement was intentional, established, and communicated from the onset of the initiative.

### 2.1. Establishing the BHDB PFLE engagement team

The BHDB steering committee tasked a working group to create a strategy for facilitating PLFE engagement throughout the initiative's life cycle. This task dovetailed with CAMH's clinical and research patient and family engagement strategy and the organizational PFLE engagement roadmap. The roadmap grounded and adapted the Carman patient engagement framework (18, 30). Moreover, four clinical and research family advisory committees were established, which included CAMH PFLE engagement facilitators and coordinators who were trained specialists in PFLE engagement.

Based on the recommendation of CAMH PFLE facilitators and coordinators, the BHDB team was invited to join various PFLE advisory committees to introduce the BHDB and recruit individuals to join the BHDB PFLE engagement team. While this opportunity attracted PFLE advisors interested in AI/ML and digital health technologies, there was no requirement to have prior knowledge or experience in these areas. As team members, the recruited three BHDB PFLE partners ensured PFLE engagement, representation, and participation in BHDB activities.

To kick off the BHDB PFLE engagement team, Terms of Reference were co-created to establish roles, goals, and a common understanding of contributions. Since BHDB partners were also members of their respective CAMH PFLE advisory committees, they were responsible for reporting BHDB developments to their respective committees and soliciting feedback when necessary. As engagement team members, the partners would provide leadership and expertise in planning and supporting the engagement strategies and activities; make recommendations on meaningful engagement and representation; join the working groups within and outside the governance structure; and participate in designing various projects. To ensure accountability, an activity-tracking tool was used to document all the projects, goals, and ideas stemming from the project while providing the team with the status of various project objectives. The following section highlights three major projects where the team identified and developed mechanisms for PFLE engagement.

### 2.2. Different types of PFLE engagement across BHDB projects

#### 2.2.1. Project 1: consultation—research and care coordination

As a first step to developing a digital platform to support clinics with the coordination of research and care, the BHDB launched a series of five interactive virtual workshops to engage stakeholders across the hospital to gather user requirements. BHDB partners invited advisors from their respective advisory committees to participate. Advisors were encouraged to attend a pre-workshop orientation session where they were briefed with information about the context, background, technical terminology, workshop expectations and outcomes.

The PFLE advisors were invited to attend multiple workshops. Their participation accounted for 21.5% (31/144) of stakeholder engagement across all five workshops. At the virtual workshops, PFLE advisors participated in breakout sessions and discussions with clinicians, scientists, and hospital administrators. Participants provided their user requirements for the digital platform and collectively prioritized the modules identified through the workshop. The PFLE engagement was critical in advocating for a patient-facing interface, ranked in the top 5 of 12 priority areas. This finding initiated the development of a patient-facing portal to be included. The clinical and patient portals are currently under development and will be integrated into a CAMH-wide digital initiative.

#### 2.2.2. Project 2: involvement — co-designing the patient trajectory dashboard

As the BHDB clinical treatment trajectory dashboard was integrated across various CAMH clinics to support clinician decision-making, there was a growing recognition that a patient-facing dashboard can also benefit patients and families. This initiated a project to co-design a patient journey dashboard and was brought to the BHDB PFLE engagement team to plan. A BHDB partner volunteered to be the project co-lead with a design student from a local university. As co-leads, they planned and conducted the human-centred design project. They contributed to developing questions for semi-structured interviews and interviewed seven PFLE advisors and three clinicians. The joint analysis of gathered information informed the creation of a wireframe prototype. A formal project report and presentation were co-developed and co-presented to participants and BHDB stakeholders. This project initiated the future integration of a digital patient journey tool in the next iteration of the CAMH patient portal.

#### 2.2.3. Project 3: partnership—BHDB governance

The BHDB PFLE engagement team operated on a partnership model with a shared decision-making process for enacting PFLE engagement opportunities. This included early engagements with the BHDB steering committee and external scientific advisory committees. However, the engagement team understood that if the goal was for a meaningful engagement at all levels, it was a natural evolution for the team to advocate for its PLFE partners to be members of the BHDB steering committee. This would enable PFLE to directly influence decision-making through their interactions with senior leadership and administrators on the committee. As a result, two PFLE partners formally joined the BHDB steering committee. To ensure a smooth transition to this new responsibility, roles and expectations were communicated, and the opportunity to have additional briefing sessions before meetings. As SC members, PFLE partners raised agenda items on the team's behalf and provided updates on PFLE engagement activities during SC meetings. It has been a positive experience for the other steering committee members and PFLE partners, with positive anecdotal feedback from all parties involved. Participation at all levels in this work has also increased the visibility of PFLE engagement, which has led to additional opportunities for PFLEs, such as co-presenting to the hospital board of directors. Moreover, this approach was lauded by the external scientific advisory committee.

## 3. Discussion—critical success factors and lessons learned

This perspective article is intended to contribute to our understanding of PFLE engagement in digital health and AI and this dynamic field of practice. While there is increasing recognition that PFLE engagement is crucial to the acceptability of AI in healthcare, engagement is predominantly overlooked in the development of AI or has a limited presence in academic literature (31–33). A recent scoping review on patient and public involvement in AI and digital health in mental health (33) found that only 5 of the 144 articles identified focused on PFLE, whereas most studies used and accessed patient and public perspectives to inform a project or co-design technologies. This paper provides some preliminary insights into other knowledge gaps in PFLE engagement in AI by reflecting on our longitudinal experiences in the context of mental health.

**Figure 1:**
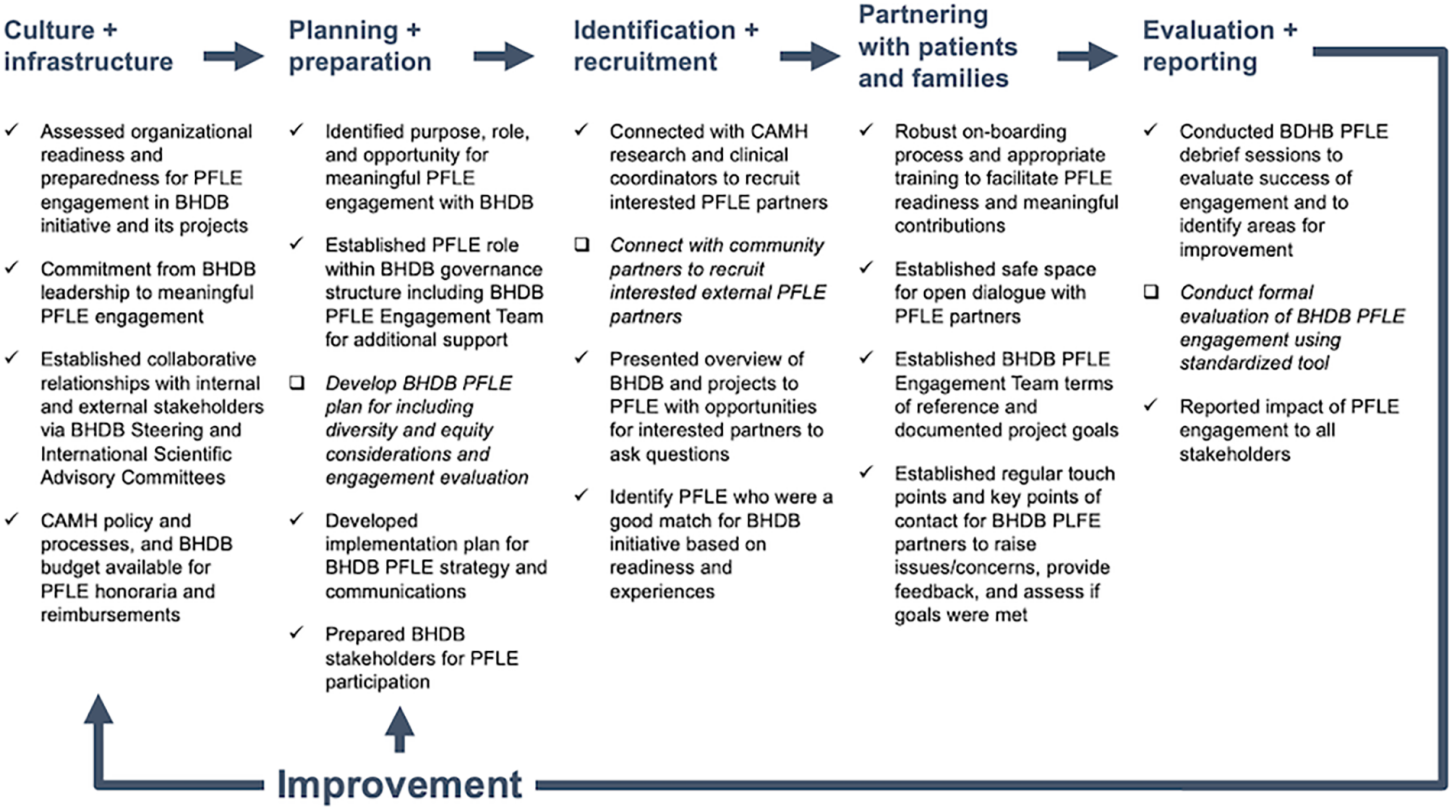
BrainHealth Databank patient, family, and persons with lived experience (BHDB PFLE) engagement process map [adapted from Shen et al. (19)]. The process map was created to provide an overview of PFLE engagement processes throughout the BHDB lifecycle. Check marks (√) indicate practices that contributed to successful PFLE engagement while check boxes (□) and italicized font indicate areas for improvement moving forward. BHDB, BrainHealth Databank; PFLE, patients, families, and persons with lived experiences.

This article also documents a whole system approach to PFLE engagement (34), where engagement considers the individual, team, and organizational implementation factors. Based on feedback from BHDB internal and external stakeholders, PFLE partners and advisors, and use case outcomes described above, our dynamic approach to PFLE engagement has been successful overall. Guided by the PFLE process map (Figure 1) (19), we highlight the factors that contributed to our success and identify areas for improvement.

### 3.1. Culture and infrastructure

The commitment at the CAMH organization, BHDB leadership, and PLFE individual level led to active PFLE engagement in various degrees across the BHDB implementation. At the organizational level, CAMH's culture and PFLE engagement infrastructure immensely benefited the BHDB. At the BHDB leadership level, the genuine commitment and support to achieve meaningful PFLE engagement at all levels of the BHDB contributed to the achievements to date. These findings align with existing literature and frameworks on developing organizational capacity for PFLE engagement (35).

We were fortunate to be situated in an environment where mobilizing PFLE engagement across all CAMH activities was an organizational priority. The organizational commitment and infrastructure aligned with the BHDB's approach to building, developing, and growing partnerships with PFLEs (36). The established clinical and research patient and family advisory committees created a community of interested PFLE experts for the BHDB to draw upon with PFLE engagement to build engagement capacity by educating and training staff and PFLEs. Institutional policy and processes for providing honoraria for PLFE participation were formalized as a requirement, acknowledging time for PFLE expertise. This organizational readiness immensely benefited BHDB's PFLE engagement plans, particularly for facilitating recruitment.

### 3.2. Planning and preparation

Readiness across the system enabled PFLE engagement at a team level. Establishing the BHDB PLFE engagement team was critical to integrating engagement across the breadth of activities in developing and implementing the BHDB. First, it connects the various project teams, committees, and the PFLE engagement community. This connection enabled the wayfinding and identification of PFLE engagement opportunities, evaluation of feasibility, and development of plans to support the identified opportunities. Secondly, having a team of on-boarded PFLE partners, facilitators, and coordinators with diverse expertise meet monthly allows flexibility and responsiveness in planning and mobilizing for engagement activities. This was especially valuable given that opportunities were often attached to short timelines and required an understanding of the complexity and breadth of the BHDB initiative.

### 3.3. Identification and recruitment

The BHDB's scale and focus on cutting-edge digital health and AI technologies have attracted PLFE participation. Opportunities to engage at various levels and with varying roles allowed us to attract and recruit advisors with different experience levels and diverse backgrounds from the respective CAMH advisories. For example, those newer to the role of PFLE advisor were more comfortable in a consultation role, while those with experience were drawn to leadership roles. PFLE readiness influenced the degree to which advisors participated in this work.

PFLE recruitment has solely been from the CAMH clinical and research patient and family advisory committees. While this has allowed us to be agile in responding to engagement opportunities with tight timelines, as experienced in case study 1, this approach has shortcomings. Recruitment was from a community of advisors supporting mental health care and research at CAMH. Much of this work was during the pandemic when recruiting from the community and clinics was difficult. Recognizing the need for a greater diversity of perspectives, the team will explore additional avenues for engagement, including reaching beyond the walls of CAMH.

A robust onboarding process is necessary to achieve meaningful engagement and ensure PFLE readiness, which requires understanding project background, context, and PFLE role. There was no requirement to have prior knowledge of digital health or ML/AI to participate. The onboarding process and engagement sessions were virtual due to the COVID-19 pandemic. While this removed the need for travel and provided greater flexibility for participation, this also created the requirement for participants to have access to technology. An option to call in and receive materials via email was provided to support those without access to stable internet.

Orientation sessions involved a project overview presentation including expectations, time commitments and anticipated outcomes, followed by a question-and-answer period, were held for each case study. For use cases 1 and 2, PFLE partners helped to co-create practical and accessible project orientation and background information materials, such as a terminology cheat sheet.

### 3.4. Partnering with PFLEs

At the PFLE individual level, a sense of understanding and appreciation of their impact on the BHDB motivated participation. Establishing a safe environment and building trust with PFLE Partners is vital for supporting meaningful engagement. One way the BHDB accomplished this was by ensuring that there was always more than one PFLE team member present during activities. PFLE partners could also contact PFLE staff facilitators and coordinators to raise any issues and concerns. These avenues minimized the risk of power differentials impacting this work.

Fostering open dialogue in these meetings was a critical prerequisite for thoughtful and meaningful PFLE engagement. To create a safe space for open dialogue, monthly BHDB PFLE team meetings provided a comfortable space for the team to check-in, share BHDB updates, learn about related projects, put forward items and recommendations to the SC and external scientific advisory committee, and, most importantly, make decisions on matters about PFLE engagement. It also ensured a common understanding of projects and objectives and allowed the team to ask questions and work through knowledge gaps and challenges.

These regular monthly team meetings also provided a consistent cadence for sharing BHDB activity updates which were critical in enabling PFLE partners to identify engagement opportunities, fostering a sense of shared accountability for this work. While BHDB updates would frequently come from the project team, other key BDHB stakeholders were invited to present to this group. Given the enormous scope of the BHDB, sharing updates is challenging and an area to improve upon. These factors echo the experiences of other PFLE engagement initiatives (37) and the literature on meaningful engagement (34).

### 3.5. Evaluation and reporting

Debrief sessions were held by either BHDB or CAMH advisory committees post-PFLE engagement to assess meaningful engagement and gather feedback. Regular reporting on PFLE engagement activities to stakeholders occurred at every BHDB SC and external scientific advisory committee meeting. Any feedback received was discussed at subsequent BHDB PFLE team meetings, and an action plan was developed accordingly. The opportunity to request additional information on any topics or issues discussed during these meetings was also provided to improve understanding of the matters discussed.

In retrospect, formal evaluations of the engagement were overlooked, which is a limitation of our work to date. Moving forward, implementing standardized evaluation tools and frameworks will help systematically evaluate the impact of each engagement and inform future engagements (38, 39). Tools and frameworks such as the Public and Patient Engagement Evaluation Tool (PPEET) (40) and Guidance for Reporting Involvement of Patients and the Public (GRIPP2) (41) would allow for more consistent evaluations and reporting, thereby improving applicability across contexts. Sharing lessons learned will be critical in advancing the collective understanding on meaningfully engaging PFLE in implementing these large initiatives, documenting how we circumnavigate emerging challenges.

## 4. Future directions and conclusion

As the BHDB continues to grow, there is an imperative to keep improving our approach to PFLE engagement. In our future engagements, we aim to increase the diversity of PFLE perspectives in our engagements and team. Moving forward, we will explore additional areas for engagement—for example, building capacity for PFLE engagement in research through the BHDB. With the eventual integration of BHDB AI use cases, PFLE engagement becomes increasingly critical to ensure the development of responsible and ethical products that meet the community's needs, especially concerning AI's appropriateness, equity and fairness, privacy, governance, and transparency. Formal evaluations of those engagements should be a foundational activity to ensure it is increasingly meaningful and impactful for the individuals, teams, and organization. By sharing how the BHDB navigated and fostered PFLE engagement, we hope this will motivate and inspire the digital health, AI, and mental health community to continue to collectively chart this path to both support advancements and implementation of novel cutting-edge digital health and ML/AI products that will improve brain health.

## Data Availability

The original contributions presented in the study are included in the article/Supplementary Material, further inquiries can be directed to the corresponding author.
